# Atlas and Abstract of the Diseases of the Larynx

**Published:** 1899-12

**Authors:** 


**Atlas and Abstract of the Diseases of the Larynx.** By Dr. L. GRÜNWALD. Edited by CHARLES P. GRAYSON, M.D. London: The Rebman Publishing Co. (Ltd.). Philadelphia: W. B. Saunders. 1898.

This little volume is by no means an atlas of the diseases of the larynx pure and simple, but, as the American editor happily and very truly says in his preface, "it exemplifies a happy blending of the didactic and clinical;" for the remarkably faithful 107 coloured figures of the laryngoscopic and histological appearances of various conditions are associated with nearly 100 pages, having a concise and clear summary of the main points to be noted and their import. The full-page plates are accompanied with very full descriptions. To advanced students and practitioners the work will prove an extremely useful handbook.

